# Nanomaterial Safety Management in Practice: Expert Perspectives on Implementation Challenges and Organizational Approaches in Singapore

**DOI:** 10.1177/10482911251357978

**Published:** 2025-07-17

**Authors:** Sriram Prasath Ramasoori Krishnan, Kavitha Palaniappan, Sally Chan

**Affiliations:** 1 5982 The University of Newcastle, Newcastle, Australia; 2 121579 Duke-NUS Medical School, Center of Regulatory Excellence, Singapore, Singapore; 3 261919 Tung Wah College, Hong Kong, Hong Kong

**Keywords:** nanomaterial safety, risk management, organizational safety culture, regulatory compliance

## Abstract

This study investigated nanomaterial safety management through semistructured interviews with 14 subject matter experts across industry, research, and regulatory domains. Expert perceptions, practical implementation of exposure controls, and barriers to effective safety management in Singapore's nanotechnology sector were examined. Thematic analysis using MAXQDA software identified 6 key themes: information management and organizational practices (28%), training and knowledge management (26%), documentation and risk management (21%), advanced manufacturing and implementation insights (14%), geographic and regulatory framework variations (11%), and measurement and characterization challenges (6%). The study identified significant variations in how organizations approach safety management, particularly in information sharing, training delivery, and control measure implementation. Technical challenges in exposure measurement and characterization emerged as critical barriers, while documentation and risk management practices varied considerably across different organizational contexts. This research contributes to nanomaterial safety management by providing insights into practical implementation challenges across diverse organizational contexts.

## Introduction

Engineered nanomaterials (ENMs) are increasingly transforming commercial and industrial applications through their unique physicochemical properties and versatile functionalities. The field has evolved from early technological optimism to established commercial implementation, with current global market valuations exceeding $30bn in 2024.^1^ While approximately 5 percentage of companies globally engage in ENM production or development, the workforce directly handling these materials remains relatively small within individual organizations. However, the increasing number of ENM applications across industries signals an inevitable increase in total ENM use and in worker exposure.^2^

This expanding integration of ENMs into manufacturing processes has raised significant concerns among occupational health experts and the scientific community regarding worker safety. While nanotechnology enables innovation in both consumer and industrial products, manufacturing facility workers face risks due to the likelihood of exposure throughout the production lifecycle, complicated by nanomaterials’ variable properties that can change dramatically with subtle alterations in their morphometrics (shape and size characteristics), surface characteristics, and medium of introduction.^3,4^ These variations significantly challenge workplace exposure assessment and the development of standardized occupational safety protocols in workplaces.^5^

Recognizing these complex exposure risks, the scientific community has initiated global research programs to identify proactively and to mitigate potential health risks in nanomaterial workplaces. While these efforts build upon foundational epidemiological studies from the 1980s and 1990s that examined worker exposure to early nanomaterials like carbon black and nanotitania, current ENM research presents distinct challenges.^6,7^ Although laboratory studies provide crucial mechanistic insights, establishing reliable safety thresholds and preventive measures requires comprehensive data from multiple sources, including workplace exposure assessments, epidemiological surveillance, and toxicological studies.^8,9^ This validation is particularly challenging due to the fragmented nature of the ENM workforce, where small worker populations are distributed across multiple countries and companies. Such distribution necessitates robust international collaboration to achieve statistically meaningful epidemiological studies of novel nanomaterials and their health impacts.

Current occupational health studies in nanomaterial contexts face multiple interconnected challenges. Primary methodological barriers stem from inadequate standardization in exposure assessment protocols and significant variability in nanomaterial metrics.^10^ This technical complexity is further compounded by limited biological understanding, particularly regarding long-term health effects and relevant biomarkers.^11,12^ Second, the workforce structure presents additional challenges, characterized by a predominance of research and development activities over large-scale production, resulting in statistically challenging small population sizes and insufficient disease latency periods for epidemiological studies.^11,13^ Furthermore, fragmented regulatory frameworks and lack of harmonized worker registration systems complicate long-term surveillance efforts. Beyond these technical and structural challenges, significant knowledge gaps exist in recognizing organizational risk perception and management across different operational contexts. Studies^14–16^ have particularly highlighted significant gaps in interpretation how organizations across different operational contexts perceive and manage nanomaterial risks, suggesting a critical need for research addressing these socio-organizational dimensions.

These intersecting challenges including methodological limitations, workforce characteristics, and regulatory fragmentation, highlight a critical need for research that extends beyond technical considerations of nanomaterial safety. While laboratory studies and exposure assessments remain crucial, the successful implementation of safety measures ultimately depends on organizational dynamics, expert engagement, and practical workplace realities. The complexity of nanomaterial risk management requires understanding both technical and socio-organizational dimensions of safety implementation.

Given these challenges and the rapidly evolving nature of nanomaterial applications, this study employs a qualitative research approach through semistructured interviews with nanomaterial safety experts in Singapore's nanotechnology sector. This research addresses a crucial gap in current literature by examining the practical challenges and organizational factors influencing nanomaterial safety management. By encompassing diverse expert perspectives within Singapore's nanotechnology sector, the study provides unique insights into safety management practices across varied organizational contexts, contributing valuable understanding to both local and global efforts in nanomaterial safety management. These insights will inform the development of evidence-based guidelines and protocols, enabling organizations to establish more effective exposure control strategies tailored to different operational contexts. Furthermore, the findings will support regulatory bodies in creating practical guidance documents while enhancing training programs and risk communication strategies based on real-world implementation experiences.

## Research Objectives

This study aims to understand:

- The perceptions of key experts who participate in controlling exposures.
- Experts’ understanding of exposure controls and the implementation practices for these control measures.
- The perceived barriers and factors to the implementation of controls in workplaces.
- The views from a regulatory perspective on controls in workplaces and the implementation of exposure limits in protecting the workers involved in handling nanomaterials.

## Methodology

Semistructured interviews were conducted with subject matter experts in nanomaterial safety and management, following ethical approval from the University of Newcastle (Reference No. H-2018-0514). The interview protocol was developed based on control measures outlined in the guidance document “Controlling health hazards when working with nanomaterials: Questions to ask before you start” published by the Nanotechnology Research Center at the National Institute for Occupational Safety and Health (NIOSH).^17^ This methodological approach was selected as optimal for several reasons. First, semistructured interviews allow for in-depth exploration of complex safety management practices while maintaining consistency across participants through a standardized protocol. Second, this format provides the flexibility to probe emerging themes and capture nuanced perspectives on implementation challenges that may not be readily apparent through structured surveys or questionnaires. Additionally, the semistructured format enables participants to share context-specific experiences and insights that are particularly valuable in understanding the practical challenges of nanomaterial safety management across different organizational settings. The interview guide included open-ended questions addressing key domains of nanomaterial safety practices: risk assessment protocols, control measure implementation, training procedures, documentation systems, and regulatory compliance strategies. Follow-up probes were employed to elicit detailed descriptions of institutional practices and perceived implementation barriers.

Purposive sampling was employed to recruit participants representing key expert groups in Singapore's nanotechnology sector. The target population included industry personnel, occupational hygienists, health and safety professionals, researchers, and government representatives with at least 5 years of experience in managing nano-TiO_2_ or related nanomaterial applications. Initial recruitment occurred through multiple channels: direct email invitations to previous study participants, distribution of information through professional organizations (OEHS Singapore, Singapore Chemical Industry Council [SCIC], Singapore Institution of Safety Officers, NIOSH, and International Occupational Hygiene Association [IOHA]), and nanotechnology networking events and conferences. Additional participants were identified through snowball sampling, where initial participants referred other qualified experts. Participants were required to have at least 5 years of professional experience in nanotechnology-related industries with direct involvement in environmental health and safety (EHS), occupational hygiene, or nanomaterial management. Those without relevant EHS-related responsibilities were excluded. Of the 30 qualified candidates identified and invited, 10 (33%) consented to participate in the recorded interviews, 4 (13.3%) explicitly declined, 1 (3.3%) agreed to complete an online survey but declined the recorded interview option, and 15 (50.0%) did not respond to the invitation. To supplement the initial recruitment, snowball sampling was implemented, yielding an additional 4 participants through referrals from initial respondents. This resulted in a final sample of 14 participants.

Participant recruitment concluded upon reaching data saturation, a methodological principle in qualitative research where additional interviews cease to yield novel themes or insights.^18^ This sample size aligns with established qualitative research practices for specialized technical domains, particularly given the participants’ high level of expertise and the relatively small population of qualified experts in Singapore's nanotechnology sector.^19^ The diversity of participants’ backgrounds, spanning industry, research, and regulatory domains, provided comprehensive coverage of the research objectives while maintaining analytical depth.

Interviews were conducted virtually between October 2023 and April 2024, with durations ranging from 26 to 64 min for each interview. All interviews were recorded with participants’ consent and conducted by a single researcher to maintain consistency. During interviews, hand-written notes were taken to capture contextual information and nonverbal cues, supplementing the audio recordings. The interview protocol was consistently followed while allowing for exploratory discussion of emerging themes.

Data analysis followed a systematic approach, beginning with verbatim transcription of all interview recordings. Transcripts were analyzed using MAXQDA Analytics Pro (version 2020), computer-assisted qualitative data analysis software that supported our content analysis approach through its coding, retrieval, and visualization capabilities. A hybrid coding strategy was employed, combining deductive codes from the interview protocol with inductive codes that emerged during analysis. Coded data were organized into a structured framework comprising major themes and corresponding subthemes, which reflected key areas such as risk management, training, documentation practices, and regulatory differences. The distribution of codes across these themes was quantified to support comparison and pattern identification across organizational contexts.

## Results and Discussion

### Participant Characteristics and Demographics

The study comprised of in-depth interviews with 14 subject matter experts in nanomaterial safety and management as presented in Table 1.

**Table 1.** Participant Profile and Roles.

Participant ID	Gender	Education	Years of experience	Current role	Location	Research and academia	Policy	Industry
Participant 01	Male	PhD	>30	Research expert specializing in multiple nanoparticle applications.	SE Asia	✓	✓	
Participant 02	Female	Master’s	15 to 20	EHS senior manager in the semiconductor industry.	SE Asia			✓
Participant 03	Male	PhD	>25	Research leader in nanomaterials, occupational hygiene and environmental risks.	Europe/SE Asia	✓	✓	✓
Participant 04	Male	PhD	>15	Academic research lead in nanomaterials applications.	SE Asia	✓		
Participant 05	Male	PhD	>40	Pioneer in occupational safety and nanotech risk management.	United Kingdom/SE Asia	✓	✓	✓
Participant 06	Male	PhD	>35	Regulatory expert in hazard communication and nanotechnology risk awareness.	United States/SE Asia	✓	✓	✓
Participant 07	Male	PhD	>30	Global research center leader in nanomanufacturing safety guidelines.	United States/SE Asia	✓	✓	✓
Participant 08	Male	Master’s	>30	Corporate director of chemical safety and international nanomaterial compliance.	Europe/SE Asia		✓	✓
Participant 09	Male	PhD	15 to 20	Occupational health specialist in nanomaterials policy and industry collaboration.	SE Asia	✓	✓	
Participant 10	Male	Master’s	>20	Certified industrial hygienist with years of experience in manufacturing nanomaterials.	SE Asia			✓
Participant 11	Female	PhD	>8	Research scientist focusing on nanomaterials.	SE Asia	✓		
Participant 12	Male	Bachelor’s	>30	Industry compliance director; established international safety standards for nanomaterial handling.	Europe/SE Asia		✓	✓
Participant 13	Male	Master’s	15 to 20	Certified industrial hygienist managing nanoparticles in EHS systems in a global manufacturing company.	SE Asia			✓
Participant 14	Male	PhD	>30	Principal researcher in nanomaterial safety; led research initiatives in nanoparticle risk assessment.	United Kingdom/SE Asia	✓		

Abbreviations: EHS, environmental health and safety; SE, Southeast.

Analysis of participant demographics revealed that a significant majority (71%) possessed more than 20 years of experience in their respective fields. The expertise distribution showed notable overlap across domains, with 43 percentage of participants having expertise in multiple sectors. The most frequent combination was industry and policy expertise, represented by 3 participants. Notably, all participants with research backgrounds also maintained expertise in at least one other domain.

### Distribution of Themes Across the Various Sectors

**Figure 1.**
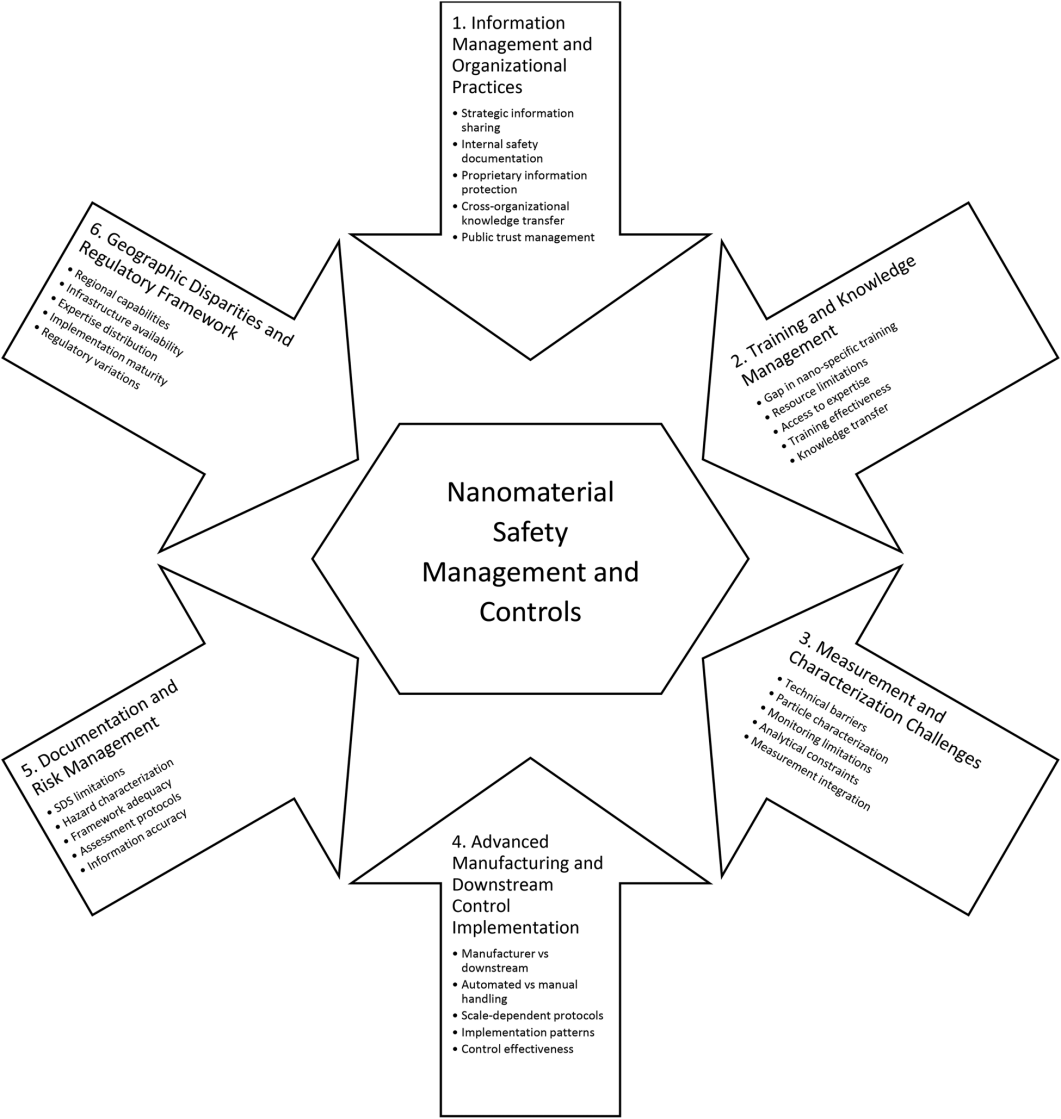
Relationship between themes and subthemes.

Table 2 presents the thematic analysis results from the 14 interviews, revealing 6 distinct themes and their corresponding subthemes. The coding process quantifies the prominence of each theme through distribution percentages, showing their contribution to total coded content and the breakdown of subthemes within each theme. Figure 1 illustrates the thematic framework and interrelationships between themes and subthemes.

**Table 2.** Theme and Subtheme Analysis.

Theme	Total coded themes and subthemes	Theme (%)	Subtheme	Code count	% of subtheme
Information management and organizational practices	57	11.40	Strategic information sharing	25	43.90
			Public trust management	13	22.80
			Cross-organizational knowledge transfer	11	19.30
			Proprietary information protection	6	10.50
			Internal safety documentation	2	3.50
Training and knowledge management	103	20.60	Knowledge transfer	46	44.70
			Gap in nanospecific training	25	24.30
			Training effectiveness	16	15.50
			Resource limitations	9	8.70
			Access to expertise	7	6.80
Measurement and characterization challenges	32	6.40	Particle characterization	14	43.80
			Measurement integration	5	15.60
			Technical barriers	5	15.60
			Monitoring limitations	5	15.60
			Analytical constraints	3	9.40
Advanced manufacturing and downstream control implementation	134	26.70	Control effectiveness	52	38.80
			Implementation patterns	51	38.10
			Scale-dependent protocols	18	13.40
			Manufacturer vs downstream	13	9.70
Documentation and risk management	80	16.00	Assessment protocols	25	31.30
			Information accuracy	22	27.50
			Hazard characterization	18	22.50
			Framework adequacy	11	13.80
			SDS limitations	4	5.00
Geographic disparities and regulatory framework	95	19.00	Regulatory variations	76	80.00
			Implementation maturity	15	15.80
			Infrastructure availability	3	3.20
			Implementation patterns	1	1.10

Abbreviation: SDS, Safety Data Sheet.

The theme and subtheme analysis demonstrated a clear emphasis on procedural elements over technical advancement, particularly evident in standardization and measurement domains. Knowledge transfer mechanisms showed consistent patterns, especially within the industry sector. Measurement challenges had minimal representation in the analyzed data. Although *Measurement and Characterization Challenges* were less frequently coded (6.4%), this theme represents a cross-cutting concern that underpins several other domains. Its relatively low frequency in the data may reflect the embedded nature of these challenges within broader discussions rather than their lack of significance—particularly given the foundational role that accurate characterization plays in risk assessment and safe handling of nanomaterials.

### Information Management and Organizational Practices in Nanomaterial Safety

Organizations implemented strict safety protocols, reflecting their recognition of nanomaterials as novel substances with distinct properties from bulk materials. Organizations demonstrated a nuanced approach to information management, with complex interplay between safety documentation requirements, the protection of proprietary information, and the need to maintain public trust. While the interviewees from larger organizations often exceeded regulatory requirements with comprehensive internal data collection, their external information sharing was carefully calibrated. This calculated approach to information dissemination is characteristic of the industrial sector, participant 06 stated,

The culture of the business, that dealt with these particles, was one that they like to have a lot of control over the information that was made available for products, I don't mean that they were trying to be secretive, more that they wanted to make sure that they could manage the distribution.

This strategic approach potentially limits the development of broader industry knowledge bases, as organizations often maintain extensive internal databases of safety information, including exposure monitoring data, incident reports, and protocol effectiveness assessments, but carefully manage the external sharing of this information and maintain it through formal channels.

The analysis reveals complex dynamics in regulatory compliance across the various industry sectors, particularly in the relationship between formal requirements and practical implementation. While existing regulatory frameworks appear theoretically sufficient, their practical implementation posed significant challenges, especially for small- and medium-sized enterprises (SMEs). Larger companies typically demonstrated more comprehensive safety measures, including engineering controls and systematic risk management approaches. In contrast, SMEs and research startups demonstrated concerning departures from the hierarchy of controls, frequently relying primarily on personal protective equipment (PPE) as their primary control measure. PPE-only approaches are unacceptable as permanent solutions for ENM exposure control and represent a critical regulatory enforcement gap requiring immediate attention.

This critical regulatory enforcement gap requires immediate policy intervention to ensure SMEs implement engineering controls regardless of organizational size. Potential solutions include mandatory engineering control requirements with government-subsidized technical assistance programs, shared-facility models where multiple SMEs can access centralized containment systems, regulatory incentives for engineering control implementation, and enhanced enforcement mechanisms that do not permit PPE-primary approaches for ENM handling. Without such interventions, regulatory frameworks should establish mandatory timelines for SMEs to transition from PPE-reliant approaches to engineering control systems with enhanced oversight during the transition period. This finding aligns with previous research findings by Gause et al^13^ and Helland et al^20^ indicating that larger companies tend to implement more comprehensive risk assessment strategies as compared to smaller ones. This challenge manifests differently across organizational contexts, with larger companies implementing comprehensive systems while smaller enterprises struggle with access to fundamental knowledge and resources. The limited availability of qualified industrial hygienists further compounds these challenges, particularly affecting smaller organizations where safety responsibilities are often concentrated in generalist roles.

The interviews findings indicate that noncompliance with safety measures is driven by practical considerations rather than a lack of awareness. Factors such as time constraints, workflow efficiency, equipment accessibility, and perceived low risk contribute to a culture where safety protocols may be deprioritized in favor of operational convenience. This prioritization of operational efficiency over safety protocols was illustrated by participant 03, who was a researcher and industry expert:

But it would explain some of these erratic behaviors, erratic in the sense of like, come on, your enclosure cost ($) 20 K and you can save one hour of work time for your workers every day for your ten workers in that room, because they don't have to put on any more the of full body gear, plus the cost of the full body gear.

This observation aligns with research on safety culture adaptation in emerging technologies.^21^ The interview findings also emphasize the value of standardized protocols and checklists, particularly for smaller organizations, with case studies and inter industry experience sharing emerging as valuable tools for improving practices. Reflecting on the role of standardized protocols, a senior safety professional, participant 12 stated:

If he's able to do the risk assessment or the risk management in a more tailored way because he has the knowledge, then it should be allowed, but otherwise the checklist is always a good start. It is a starting point or a minimum at least what someone should do. Yeah, it's a minimum.

This aligns with Powers et al’s^22^ emphasis on structured “data dialogues” and cross-sector collaboration, where knowledge sharing and standardized protocols can help bridge the gap between research findings and practical implementation, particularly benefiting smaller organizations that lack resources for comprehensive nanomaterial safety programs.

### Gaps in Training and Awareness

The interview data also revealed lack of nanomaterial-specific training and awareness. A recurring theme among academic researchers was the misconception that general safety guidelines adequately cover nanomaterial handling. This misconception was highlighted by participant 01 who asserted

General safety is given and it is expected that under the general safety guidelines, this is also covered because there is a school of thought that safety is safety.

This reliance on generalized safety training often stems from a lack of resources to develop and deliver specialized nanomaterial safety programs, leaving personnel ill-equipped to handle the unique hazards associated with these materials.

Another significant finding indicates that SMEs face substantial barriers in accessing foundational knowledge and resources essential for appropriate nanomaterial handling protocols. This challenge is further exacerbated by the limited availability of qualified industrial hygienists specializing in nanomaterial safety. Current training methodologies demonstrate a notable limitation in their tendency to apply generic safety protocols rather than addressing nanomaterial-specific handling requirements. This observation corroborates^23^ findings regarding the necessity for specialized nanomaterial safety training protocols. The gap between theoretical guidelines and practical implementation was highlighted by participant 10, who emphasized:

Unless social economic study is being done on a full scale…establishing guidelines for either it is a nanoparticle or it is for any other limits … it might not really be implemented at the floor level, it could be there in the paper, but it might not be practically workable.

The perspectives gathered from the interview participants, aligns with previous research on occupational health and safety training effectiveness.^9,24^ These findings suggest that effective nanomaterial safety management requires a multifaceted approach encompassing technical considerations, implementation feasibility, workforce engagement, and socioeconomic dimensions.

### Measurement and Characterization Challenges in Practice

The technical challenges of measuring and characterizing nanomaterial exposures emerged as a fundamental concern across organizational contexts. The difficulty in distinguishing nanoscale fractions in complex exposure scenarios presents significant technical barriers to effective risk assessment. Speaking about the core technical barriers, participant 07 explained: “You never really can tell while you're on site what fraction of the airborne particulate material is nanoscale or nano size.” Expanding on these measurement difficulties, participant 13, who is also a certified industrial hygienist, highlighted similar concerns:

There was (is) no clear guidelines on those compounds. Whether it needs to be considered as a nanoparticle or it needs to be considered as a respirable particulate … there were a lot of gaps both in understanding whether it is a nanoparticle, second one in collection of the samples and having the appropriate analytical methods.

Adding an industry perspective, participant 02, working in the semiconductor industry also acknowledged persistent challenges in nanoparticle monitoring, noting “there's a lot of unknowns related to nano particles … often we lack information, lack of measuring data.” This uncertainty in particle characterization creates cascading challenges for exposure assessment and control measure selection. This limitation necessitates sophisticated analytical approaches combining multiple measurement techniques, yet such comprehensive analysis remains challenging to implement in practice.

To address this challenge, organizations increasingly adopt a hybrid approach that combines real-time monitoring with traditional sampling methods. This evolving approach to exposure monitoring, participant 06 elaborated, “We would work with them on the difference between the different levels of activity—doing nothing, implementing a total enclosure and process control, or something in between.” While this hybrid approach offers a more comprehensive assessment of exposure, it requires significant expertise and resources to implement controls effectively. This need for specialized knowledge and resources is particularly pronounced in smaller organizations, as noted earlier in the discussion, where resource constraints can limit the ability to effectively adopt and maintain such advanced exposure assessment strategies.

### Advanced Manufacturing and Implementation Insights

A notable contrast emerged between manufacturers and downstream users. Manufacturing environments typically operated with well-controlled, closed systems where nanomaterial exposure points were limited primarily to loading and unloading operations. However, as materials moved downstream, handling environments became progressively more open and complex. Illustrating this downstream complexity, participant 12 noted: “when you go to downstream use, you're going closer step by step to consumer product,” noting that these operations often involve “smaller companies, medium-sized companies, sometimes also micro-size companies,” who frequently lack the resources and expertise of advanced larger manufacturers.

The semiconductor industry provides valuable insights into this aspect of automated advanced manufacturing environments. The industry's multilayered safety approach, including sophisticated control system combines containment strategies with comprehensive monitoring. Describing their monitoring approach, participant 02, from the semiconductor industry noted:

There will be a leak detector(s) that install to the factory level, and it will link to a SCADA (Supervisory Control and Data Acquisition) system, okay, whereby it's fully monitored 24 hours, and if there's any potential leak, okay, then it will be handled.

This sophisticated approach contrasts sharply with implementation patterns in other sectors, particularly research institutions. Contrasting with this sophisticated approach adopted in industries, participant 09, an academic, reflected on the evolution of safety practices in research settings “in the past few years, because of growing awareness … we have established safety guidelines for handling of these kind of materials.” However, implementation remains inconsistent, particularly for smaller quantities and routine operations. This informal approach to small quantities was illustrated by participant 11 who disclosed, “when handling large amount … we did it inside the fume hood. But whenever while running the experiments, I needed only, like, 5 mg… I just did it outside.” This pattern was observed across multiple interviews with researchers, where formal controls are often reserved for larger-scale handling while smaller quantities receive less rigorous protection. This disparity highlights broader challenges in risk perception and control implementation across different operational contexts in laboratories.^25,26^ The participant quote illustrates risk perception and behavioral patterns in safety implementation—specifically how researchers modify their practices based on quantity handled. This finding contributes to understanding organizational safety culture and decision-making processes, which was the focus of our investigation.

**Figure 2.**
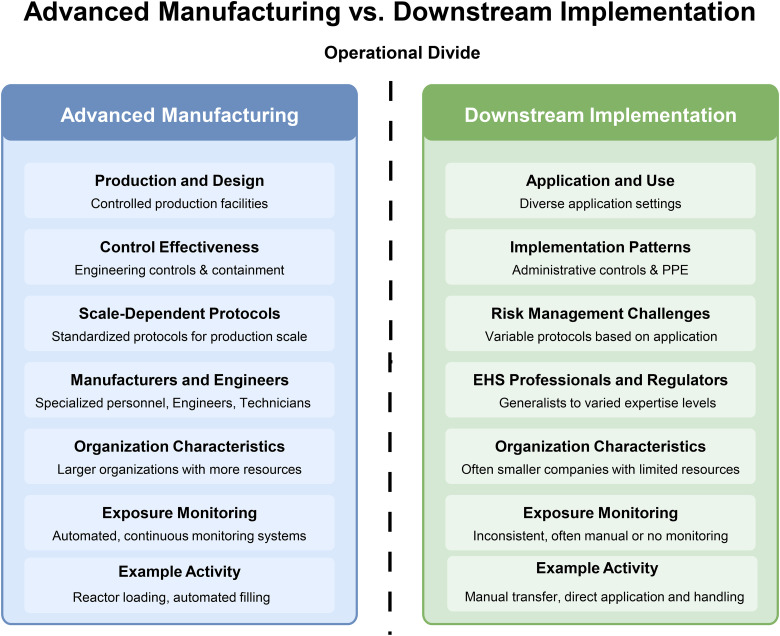
Operational divide affecting nanomaterial safety across the value chain.

A notable contrast emerged between manufacturers and downstream users in nanomaterial safety management approaches is highlighted in Figure 2. Manufacturing environments typically utilize well-controlled, closed systems, with exposure risks largely confined to specific operations such as loading and unloading. In contrast, downstream handling environments are more open, variable, and context and company culture dependent. This divide spans multiple dimensions—including control strategies, personnel expertise, and exposure monitoring practices. Advanced manufacturing settings often employ engineered controls and sophisticated monitoring technologies, whereas downstream contexts tend to rely more on administrative protocols and PPE. These differences are shaped by organizational capacity and resource availability, with larger manufacturing firms better positioned to implement integrated safety systems compared to the smaller, often less-resourced entities involved in downstream applications.

### Documentation and Risk Management Implementation

The implementation of safety documentation systems presents specific challenges across these organizational contexts. A fundamental disconnect exists between traditional documentation frameworks and the unique properties of nanomaterials. Addressing these documentation limitations, participant 06 observed: “the protocols that people use are probably not always attuned to new ideas and new awareness about potential risks.” This gap is especially evident in the development of Safety Data Sheets (SDS) for nanomaterials, where existing frameworks struggle to capture nanospecific risks. Interview data revealed persistent issues in accurately characterizing nanomaterial hazards within existing SDS frameworks. As participant 09 explained this limitation: “[the hazard characterization] is based on the SDS provided by companies, [but] the information there is not very comprehensive,” underscoring the inadequacy of current documentation practices. These challenges are particularly pronounced when dealing with materials that exist in both bulk and nano forms, where traditional hazard classification systems may not adequately capture the full spectrum of risks.^27^ In recognition of these persistent documentation challenges, recent efforts to standardize nanomaterial SDS preparation have emerged, such as ISO/TS 13329-2024 “Nanomaterials—Preparation of Safety Data Sheets (SDS)” which provides detailed guidance specifically tailored to nanomaterial characterization and hazard communication.^28^ Despite these advances in standardization, record-keeping requirements and documentation remain essential regulatory tools, though verification of compliance remains challenging.^29^

### Evolution of Control Strategies and Resource Constraints

Organizations demonstrate varying approaches to control hierarchy implementation, largely influenced by their size and available resources. Larger manufacturing companies typically implement sophisticated, multilayered control strategies, investing heavily in engineering controls and monitoring systems. Describing larger organizations’ capabilities, participant 13 noted, “if it is going to be a kind of fully enclosed environment … engineering controls do work well.” These larger organizations maintain dedicated safety personnel and comprehensive training programs, often exceeding regulatory requirements.

In contrast, smaller companies face significant challenges due to resource constraints. These limitations manifest in multiple areas, from insufficient funding and technical expertise to inadequate staffing for safety management and limited capacity to invest in control measures. The resource constraints of smaller companies were captured by participant 12 who pointed out that “The greatest risks of worker exposure are in very small companies,” where safety responsibilities are often concentrated in a single individual who “has to cover everything.”

This resource disparity creates a 2-tiered system of safety management: larger organizations with robust, systematic approaches, and smaller entities relying primarily on basic administrative controls and PPE.

### Effectiveness of Control Measures

The study reveals that while nanomaterial exposure control principles align with conventional dust management approaches, they necessitate more rigorous protocols and specialized methodologies. Engineering controls, particularly enclosed systems and negative pressure environments were identified as most effective control measures in manufacturing settings of larger companies. Another significant finding indicates heightened exposure risks along the supply chain, with downstream users encountering more challenging open handling scenarios. This finding aligns with previous research on the increasing risk profiles across nanomaterial supply chains.^30,31^

Safer by design (SbD) principles, along with the hierarchy of controls emerged as a priority among industry and policy experts, who advocated for safety integration from the development phase to downstream uses. While researchers did not focus on this aspect, SbD aligns with the “safe and sustainable by design” framework.^21^ The experts also identified implementation challenges, emphasizing the need for solutions that integrate into operations without creating excessive burden, particularly regarding resource allocation and the standardization of safety metrics across different development stages.

Validating the effectiveness of control measures through quantitative assessment presents challenges that span both technical and economic considerations. Organizations must develop robust protocols for evaluating engineering controls while managing practical constraints. Highlighting the role of technical expertise in cost management, participant 03 explained, “If they had the opportunity to consult with skilled engineering control specialists, they could implement effective controls with minimal cost and difficulty.”

However, despite their theoretical benefits, engineering controls often face operational hurdles. Participant 08 pointed out challenges with inadequately implemented engineering controls, “ ‘if the local exhaust ventilation is not properly implemented … then it becomes similar to relying on personal protective equipment (PPE)—the comfort level and user compliance become key concerns rather than the engineering control providing reliable protection.” These practical challenges including maintenance of the controls often result in the diminishing effectiveness of containment systems, with participant 01 suggesting, “those engineering controls slowly lose their value … and it just remains as a box rather than a containment box.” This highlights the gap between ideal control strategies and their real-world implementation.

The feasibility of implementing SbD strategies varies significantly across regions, influenced by local resource availability and technical capabilities. As participant 08 said “it's about having good, available and understandable solutions that people can apply,” highlighting the need for practical approaches that consider local constraints. These regional variations in resource availability and technical capacity create additional complexities in establishing standardized safety management practices, particularly for organizations operating across multiple jurisdictions.

The interviews highlight the critical intersection between technical capability and economic feasibility in implementing effective control measures.^32^ Organizations frequently struggle to establish standardized protocols that balance the need for accuracy with the constraints imposed by resource limitations and operational realities. This dichotomy between technical objectives and economic constraints further complicates the implementation of effective safety measures.

### Geographic Disparities in Nanomaterial Risk Management Implementation

The research revealed significant geographic variations in nanomaterial risk management capabilities, with notable disparities between developed and emerging economies. Organizations in North America and Europe demonstrated more sophisticated risk management approaches, characterized by robust engineering controls, comprehensive monitoring systems, and standardized safety protocols. This geographic differentiation was explicitly acknowledged by participant 05 who shared his experience: “The situation is probably different in Singapore than in Europe, and it's different in the United States, and it's different in China.”

Based on the views shared by the interviewees in the European and North American contexts, organizations exhibited enhanced technical competencies and more systematic risk management approaches, particularly evident in their implementation of hierarchical control measures. The influence of established regulatory frameworks, notably the European Union's (EU) Registration, Evaluation, Authorisation and Restriction of Chemicals (REACH) regulations, emerged as a significant driver of comprehensive risk management practices.

Conversely, Southeast Asian organizations demonstrated varying levels of risk management maturity, with significant gaps in analytical capabilities and technical expertise. The research identified several region-specific challenges:

- Limited access to sophisticated analytical infrastructure, necessitating dependence on international laboratories.
- Significant gaps in technical expertise required for comprehensive risk assessment.
- Less developed regulatory frameworks and enforcement mechanisms.
- Inconsistent implementation of engineering controls and monitoring systems.

These regional disparities were particularly evident in analytical capabilities of the laboratories managing the analysis of nanoparticles. As participant 10 noted a limitation regarding Southeast Asian laboratory facility, “So, for nanoparticle exposure assessments … laboratories with which are accredited [are] only in abroad (overseas)” highlighting limitations in local analytical capacity. This reliance on international laboratories for advanced analysis introduced additional barriers to effective risk management, including increased operational costs, extended response times, and potential gaps in monitoring capabilities.

### Regional Variations and Regulatory Framework Implementation

The implementation of safety management systems reveals significant regional disparities, particularly in analytical capabilities and regulatory oversight. Organizations operating under the EU's REACH framework demonstrate more standardized and comprehensive approaches, benefiting from clear regulatory guidelines. As participant 08 noted, “people who do business around the world tend to follow it [REACH],” indicating its emergence as a de facto global standard.

The regulatory interpretation across regions demonstrates a hierarchical approach, where companies typically look first to national occupational safety bodies, then environmental agencies, and finally research institutes. As participant 07 who is also a regulatory expert explained:

Companies look for regulatory guidance and enforcement out of the Occupational Safety and Health Administration (OSHA) … [but] doesn't have any permissible exposure limits for any nanoscale materials.

This regulatory gap creates significant implementation challenges across different jurisdictions. The disparity in testing and monitoring capabilities between regions further affects organizations’ ability to validate their safety measures effectively. This challenge is particularly acute in developing regions, where limited local analytical capacity often necessitates reliance on international testing facilities, adding complexity to data interpretation and protocol adaptation.

### Regulatory Framework Evolution and Implementation Challenges

The hierarchical approach to regulatory guidance creates additional complexities, as companies navigate multiple layers of oversight from occupational health and safety agencies, and research institutes. Senior experts from occupational safety organizations noted that while companies seek regulatory guidance and enforcement from occupational safety authorities, there is a lack of established permissible exposure limits for nanoscale materials. The same issue is applicable for Singapore's context as well where nanomaterial exposure limits are not applicable for enforcement.

The research reveals complex regulatory challenges that extend beyond basic compliance issues. A fundamental disconnect exists between policy frameworks and practical implementation capabilities, particularly in developing regions. Addressing these practical limitations, participant 13 emphasized: “It is not because we do not have legislation. The problem is that the implementation doesn't work.” This implementation gap manifests differently across jurisdictional contexts, with organizations in developing regions facing challenges in analytical capabilities and technical expertise. He added a “huge lot of work needs to be done for the basic exposure compliance. And then the nanoparticles come in the next one … it is long way to go.” This contrasts sharply with European contexts, where participant 05 stated “Europe has some pretty grand ambitions towards essentially elimination of harmful chemicals by 2030,” highlighting significant regional disparities in regulatory advancement.

Despite these challenges and contrasts, the interviews identified positive developments in international regulatory cooperation, with agencies sharing information through dossier systems to avoid duplicating existing information. However, challenges persist in harmonizing approaches across different jurisdictions. Multinational organizations operating internationally try to navigate varying regulatory requirements, creating additional complexity in compliance efforts.

### Future Directions

An emerging trend that was identified through the interviews was the shift from treating nanomaterials as special cases toward more integrated approaches to chemical safety management. This progression in regulatory thinking suggests that a more holistic approach may be more effective than nanomaterial-specific regulations. However, the experts emphasized that this transition requires better exposure data and practical evidence to inform decision-making and policy development. As participant 13 emphasized, “Unless social economic study is being done on establishing these limits or establishing guidelines … it might not be practically workable,” highlighting the need for regulatory approaches that consider both technical requirements and practical implementation constraints. The research indicates that current frameworks must evolve beyond focusing solely on toxicology to consider actual exposure scenarios and practical implementation challenges in different operational contexts.

The minimal representation of measurement challenges (*Measurement and Characterization Challenges*), despite their fundamental role in safety assessment, indicates significant gaps between technical capabilities and operational requirements in nanomaterial safety management.^33^ While not frequently emphasized as a standalone concern, measurement and characterization issues appear to be embedded within broader themes such as implementation and risk documentation. These findings suggest structural patterns in safety management prioritization, where organizations focus on procedural implementation mechanisms over technical innovation. This is reflected in the emphasis on control protocols and knowledge dissemination rather than measurement advancement. The distribution across themes indicates ongoing transitions in safety management approaches, particularly in standardization and operational integration.

This shift in approach is particularly evident in the contrasting regulatory philosophies between regions. While the EU has developed explicit regulatory frameworks that formally distinguish nanomaterials from their bulk counterparts,^34^ the United States Environmental Protection Agency (EPA) implements a more nuanced approach.^35^ Although the text of the US Toxic Substances Control Act does not explicitly differentiate between nanoscale and bulk forms,^35^ in practice the EPA administers nanomaterial regulations through specialized provisions, including information gathering requirements and premanufacture notifications specific to nanoscale materials.^35,36^ These differing regulatory implementation strategies reflect deeper variations in risk governance philosophies between regions.

Beyond these regulatory distinctions, the variation in scientific risk perception is shaped by researchers’ value systems, philosophical assumptions, and epistemologies. Studies show that factors such as disciplinary background, organizational affiliation, and professional experience influence risk assessments. This is particularly evident in nanotechnology, where a divide exists between “upstream” scientists (engineers, chemists) who focus on developing nanomaterials and “downstream” scientists (toxicologists, public health experts) who assess their risks. Studies have found that upstream scientists tend to view risks as minimal and focus on opportunities, while downstream scientists recognize both benefits and significant risks as highlighted by.^37^ These behavioral aspects of risk perception affect policy and regulatory frameworks, as seen in the EU and United States' differing approaches to nanomaterial regulation in Southeast Asia.

## Study Limitations

While this study provides valuable insights into nanomaterial safety management practices in Singapore, several limitations should be acknowledged. Due to confidentiality commitments made during participant recruitment, we did not collect or record specific organizational size metrics that would allow for precise categorization of participants as representing SMEs versus large companies. This approach was deliberately taken to maximize participant anonymity in Singapore's relatively small nanomaterials sector, where organizational identification might be possible if specific size characteristics were reported. While participants described their organizational contexts in general terms during interviews, revealing representation from research institutions, regulatory bodies, multinational corporations, and specialized firms, the absence of precise organizational size metrics limits our ability to quantitatively examine relationships between organizational size and safety management practices.

The final sample size of 14 participants, though sufficient for thematic saturation within our purposive sampling framework, necessitates caution when generalizing findings. Rather than claiming broad representativeness, our results should be considered as revealing important patterns and considerations that warrant further investigation with larger, more diverse samples. The qualitative insights generated provide valuable depth that complements existing survey-based research but would benefit from future mixed-methods approaches that combine in-depth interviews with broader quantitative assessment across a larger population of organizations.

Additionally, the study's focus on Singapore, while providing important regional perspective, may limit the applicability of some findings to other geographic contexts with different regulatory frameworks, technical resources, and safety cultures. Future research should expand geographic scope while maintaining methodological rigor to develop more comprehensive understanding of nanomaterial safety management across diverse global contexts.

This study also did not include direct worker perspectives or union representatives. In Singapore's occupational safety framework, the responsibility for regulatory compliance and safety implementation lies with employers under the Workplace Safety and Health Act. Workers are not expected to interpret evolving regulations, and labor unions such as those affiliated with the National Trades Union Congress do not function as independent advocates or representatives in occupational health and safety governance. While this aligns with the institutional structure relevant to our research focus, the exclusion of frontline worker perspectives remains a limitation. Future studies should incorporate these voices, particularly in jurisdictions where labor organizations play a more central role in workplace safety oversight.

## Conclusion

The study analyzed nanomaterial management practices through in-depth interviews with experts in EHS, senior executives, researchers, and policy development specialists. The experts’ insights during the interviews indicate that future developments in this field should focus on bridging the gap between policy and practice, particularly for smaller organizations, while maintaining a balance between safety requirements and operational efficiency. Furthermore, the development of more sophisticated exposure monitoring and assessment tools appears crucial for advancing evidence-based safety management practices. The analysis revealed significant patterns in safety protocol implementation, organizational approaches to risk management, and challenges in establishing effective safety practices across different institutional contexts. These findings provide valuable insights into the current state of nanomaterial safety management and highlight critical regulatory implementation challenges.

In Singapore, nanotechnology risk assessment and regulation have evolved through a distinct “reference-based” approach. Drawing on insights from 14 experts across Singapore, Southeast Asia, Europe, the United Kingdom, and the United States—with 57 percentage holding leadership roles in nanomaterial safety initiatives—this study revealed that Singapore strategically adapts established frameworks from the EU and United States while considering local capabilities and requirements. This adaptive approach aligns with previous research findings^38^ that emphasize the critical role of regional contexts in developing effective safety management strategies. Unlike the more prescriptive approaches found in Western regulatory systems, Singapore and Southeast Asia in general demonstrates greater flexibility in implementation while maintaining core safety principles.

The study illustrates the potential for emerging economies in Southeast Asia to pursue alignment with international safety standards, though current implementation—particularly among SMEs—often falls short of appropriate control practices. Future policy in Southeast Asia should focus on regional knowledge sharing, standardized training curricula, and public–private partnerships to address remaining gaps in nanomaterial safety management. For example, Singapore's Workplace Safety and Health Council collaborates with the SCIC to develop sector-specific training and guidance. The effectiveness of such partnerships in improving workplace safety outcomes warrants further evaluation, particularly through direct assessment of worker experiences and safety improvements. While such public–private initiatives play a supportive role, they must be accompanied by mandatory regulations that set enforceable safety baselines for all organizations. This tailored approach is essential for addressing the unique operational divide between advanced manufacturing and downstream implementation observed in Singapore's industrial ecosystem, where limited resources and technical expertise often constrain smaller organizations’ ability to implement comprehensive safety protocols.
